# Effect of Jaw Clenching on Balance Recovery: Dynamic Stability and Lower Extremity Joint Kinematics after Forward Loss of Balance

**DOI:** 10.3389/fpsyg.2016.00291

**Published:** 2016-03-11

**Authors:** Steffen Ringhof, Thorsten Stein, Daniel Hellmann, Hans J. Schindler, Wolfgang Potthast

**Affiliations:** ^1^BioMotion Center, Institute of Sports and Sports Science, Karlsruhe Institute of TechnologyKarlsruhe, Germany; ^2^Department of Prosthodontics, Dental School, University of HeidelbergHeidelberg, Germany; ^3^Research Group Biomechanics, Institute for Mechanics, Karlsruhe Institute of TechnologyKarlsruhe, Germany; ^4^Institute of Biomechanics and Orthopaedics, German Sport University CologneCologne, Germany; ^5^ARCUS Clinics PforzheimPforzheim, Germany

**Keywords:** postural control, balance recovery, dynamic stability, joint kinematics, jaw clenching, craniomandibular system, biting

## Abstract

Postural control is crucial for most tasks of daily living, delineating postural orientation and balance, with its main goal of fall prevention. Nevertheless, falls are common events, and have been associated with deficits in muscle strength and dynamic postural stability. Recent studies reported on improvements in rate of force development and static postural control evoked by jaw clenching activities, potentially induced by facilitation of human motor system excitability. However, there are no studies describing the effects on dynamic stability. The present study, therefore, aimed to investigate the effects of submaximum jaw clenching on recovery behavior from forward loss of balance. Participants were 12 healthy young adults, who were instructed to recover balance from a simulated forward fall by taking a single step while either biting at a submaximum force or keeping the mandible at rest. Bite forces were measured by means of hydrostatic splints, whereas a 3D motion capture system was used to analyze spatiotemporal parameters and joint angles, respectively. Additionally, dynamic stability was quantified by the extrapolated CoM concept, designed to determine postural stability in dynamic situations. Paired *t*-tests revealed that submaximum biting did not significantly influence recovery behavior with respect to any variable under investigation. Therefore, reductions in postural sway evoked by submaximum biting are obviously not transferable to balance recovery as it was assessed in the present study. It is suggested that these contradictions are the result of different motor demands associated with the abovementioned tasks. Furthermore, floor effects and the sample size might be discussed as potential reasons for the absence of significances. Notwithstanding this, the present study also revealed that bite forces under both conditions significantly increased from subjects’ release to touchdown of the recovery limb. Clenching the jaw, hence, seems to be part of a common physiological repertoire used to improve motor performance.

## Introduction

Postural control is crucial for most activities of daily living, and comprises the neuromuscular control of postural orientation and postural equilibrium; the latter is commonly referred to as balance. Whereas postural orientation involves the positioning of the body’s segments in space with respect to gravity, postural equilibrium delineates the ability to control the center of mass (CoM) within the base of support (BoS) (Woollacott and Shumway-Cook, 2002; Macpherson and Horak, 2013).

The main function of the postural control system is to maintain stability, and thus to prevent any falls resulting from internal or external forces (Horak and Macpherson, 1996). However, due to the large variety of structures involved in this complex sensorimotor process, falls are common and serious events, potentially leading to severe injuries or even to death. Most of these falls occur during locomotion, such as tripping or slipping while walking (Blake et al., 1988). Investigations on postural control and fall related events, hence, are a particular issue of concern – for the scientific community, for the public health care system, and also for the fall-prone persons and patients themselves.

Do et al. (1982) were the first to introduce an experimental paradigm for assessing recovery behavior during forward loss of balance. This paradigm, in which subjects are suddenly released from a static forward lean angle, is still frequently used (Wojcik et al., 2001; Karamanidis and Arampatzis, 2007; Arampatzis et al., 2008; Karamanidis et al., 2008; Curtze et al., 2010; Barrett et al., 2012; Carty et al., 2012; Cheng et al., 2014; Graham et al., 2014), and has been shown to evince postural deficits in diverse populations. In this context, force potential of leg extensor muscles (Karamanidis et al., 2008), effective control of the whole body CoM (Barrett et al., 2012), as well as step length and step velocity (Carty et al., 2012) have been identified as important variables for dynamic postural stability.

In recent years, several studies reported on the potential benefits of jaw clenching on human postural control. Thereby, a significant decrease in center of pressure (CoP) displacements induced by submaximum bite forces has been revealed by posturographic analyses (Hellmann et al., 2011; Ringhof et al., 2015b). These reductions in postural sway were accompanied by decreased sway of cranial body segments (Ringhof et al., 2015b), concomitant with alterations in muscular co-contraction patterns and systematic reductions in joint motions of the lower extremities (Hellmann et al., 2015). Modulation of somatosensory input, particularly for the neck muscles (Abrahams, 1977), and facilitation of human motor system excitability (Boroojerdi et al., 2000) were suggested to be the main causes for these improvements. In addition, facilitating effects on ankle extensor and flexor muscles (Miyahara et al., 1996; Takada et al., 2000), concomitant with attenuated reciprocal Ia inhibition from the pretibial muscles to the soleus muscle (Takada et al., 2000), which have been reported as a result of concurrent jaw clenching activities, might have contributed to the abovementioned stabilizing effects. Neuroanatomical connections and projections of the trigeminal nerve to structures associated with postural control (Ruggiero et al., 1981; Buisseret-Delmas et al., 1999; Devoize et al., 2010) are thought to form the basis for these findings.

Whereas the effects of jaw clenching on static postural control are merely consistent, there is no clear evidence as to whether dental occlusion in general affects postural sway; and also the mechanisms supporting this potential effect are still debated, and far from having reached a consensus (Cuccia and Caradonna, 2009; Michelotti et al., 2011; Manfredini et al., 2012). On the one hand, there are several studies in which significant sway reductions were observed, depending on the relative position of the mandible. Specifically, CoP displacements were found to be significantly decreased when the mandible was in symmetric centric relation as compared to intercuspal or lateral occlusion (Gangloff et al., 2000; Bracco et al., 2004; Sakaguchi et al., 2007). Contradictory results are provided by Ferrario et al. (1996), reporting that postural sway was not significantly influenced by five dental positions, either in healthy women or in women with temporomandibular disorders and asymmetric malocclusion. Perinetti (2006, 2007) confirmed these findings in terms of non-significant differences between intercuspidation and mandibular rest positions under eyes open and eyes closed conditions, disputing any relationship at the posturography level between dental occlusion and body sway. Some of this work has been criticized, however, primarily because of weak experimental designs and lack of control conditions. Moreover, in most of the publications, unfortunately, descriptions of the experimental design are inadequate. Some of the weak points are the lack of information concerning the generated bite forces and the mandibular positions during the experiments. In particular, when assessing the impact of dental occlusion on postural control, the actual oral motor activity mostly remained unknown. Furthermore, there is no international consensus about the definition of a physiological centric jaw relation (Keshvad and Winstanley, 2000). The common used phrase of symmetric positioning of the mandible in centric relation is, thus, not meaningful, and the jaw positions as experimental conditions are not comparable (Hellmann et al., 2015).

In conclusion, the contradictory reports merely are a consequence of diverse, potentially affecting experimental conditions and/or task instructions. The findings concerning the effects of jaw clenching on postural control are mostly consistent, however. Notwithstanding this, previous studies exclusively focused on the influence of force-controlled biting on postural sway under static conditions, i.e., upright unperturbed stance. To the best of the authors’ knowledge, there are no reports describing the effects of jaw clenching on postural stability in dynamic situations; which is much more related to the risk of falling than static postural control (Rubenstein, 2006).

The purpose of this study, therefore, was to investigate the effects of submaximum jaw clenching on dynamic stability and lower extremity joint kinematics during balance recovery after forward loss of balance. This methodological approach comprises components of postural control, muscular strength and reaction time. As clenching of the jaw was shown to significantly improve reflex facilitation (Miyahara et al., 1996; Takada et al., 2000), static postural control (Hellmann et al., 2011, 2015; Ringhof et al., 2015b), force production, and rate of force development (Forgione et al., 1991; Hiroshi, 2003; Ebben et al., 2008), it was hypothesized that submaximum biting would lead to improved balance recovery in terms of increased dynamic stability. We also hypothesized a concomitant decrease in joint flexion angles of the knee and hip of the recovery limb at touchdown as well as during the subsequent stance phase.

## Materials and Methods

### Subjects

Twelve healthy young adults, 10 males and 2 females, with a mean age of 21.8 ± 1.8 years (height: 1.78 ± 0.04 m; mass: 72.85 ± 2.35 kg) participated in the study. All participants were naïve to the experiments, and presented with full dentition (except for third molars) in neutral occlusion. None of them had any self-reported muscular or neurological diseases that could have affected their ability to perform the experiments.

The study was approved by the Ethics Committee of the German Sport University Cologne (no. 38/12), and written informed consent was given by all subjects.

### Experimental Procedure

To evaluate the effects of jaw clenching on balance recovery, a crossover design was applied. The experimental design included a balance recovery task in the form of a simulated forward fall, and two oral motor tasks: force-controlled biting and non-biting. The order of oral motor tasks was counterbalanced across the subjects, i.e., half of the sample started with force-controlled biting, whereas the others first performed the non-biting control condition.

#### Oral Motor Tasks

Force-controlled biting (FB) was conducted at submaximum bite forces of 150 N, corresponding to mean individual maximum voluntary contraction of the masseter of 15.07 ± 4.47%. This bite force is in accordance with previous experiments (Hellmann et al., 2011), revealing that submaximum biting significantly affected postural sway in upright unperturbed stance. To monitor the bite forces, a hydrostatic system consisting of liquid-filled pads fixed to the maxilla was used. Biting on the pads resulted in increased hydrostatic pressure, which was presented to the subjects as numerical real-time feedback on a display positioned directly in front of them. Detailed information on the hydrostatic system and the oral splints can be obtained from Hellmann et al. (2011) and Ringhof et al. (2015b).

In the non-biting control condition (NB) the oral device was worn as well, but the subjects were asked to keep their mandible in a resting position, that is consciously applying no bite force, and monitoring this condition by looking at the feedback screen.

#### Balance Recovery Task

Forward falls were simulated by an experimental approach that has been previously reported by Do et al. (1982), and Karamanidis and Arampatzis (2007). Within this test, subjects were instructed to attempt to recover balance by taking a rapid single step after being suddenly released from an inclined forward posture (Wojcik et al., 2001; Karamanidis et al., 2008).

**FIGURE 1:**
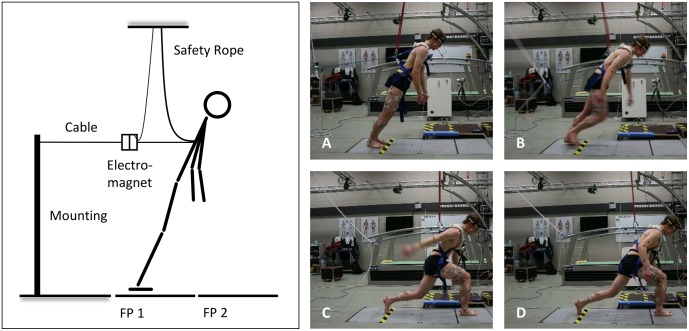
**Schematic illustration of the experimental setup (left) and the analyzed time points (right): release of the subject **(A)**, toe-off from the ground of the recovery limb **(B)**, touchdown of the recovery limb **(C)**, and 500 ms after touchdown **(D)**; FP, force plate**.

In the present study, the forward-inclined position was attained by a horizontal cable that was attached to a safety harness worn by the subjects around the trunk. At the other end, the horizontal cable was connected to an electromagnetic system, which could be manually released by the investigators (**Figure 1**). To avoid any injuries resulting from falls, the safety harness additionally was attached to a ceiling-mounted rope, which prevented contact of any body part, other than the feet, with the ground (Karamanidis et al., 2008).

At the beginning of each trial, the subjects stood barefoot with both feet on a force plate (AMTI, model BP600900, 1,000 Hz; Advanced Mechanical Technology, Watertown, MA, USA), and were then moved in a forward-inclined position. The angle of this leaning position was individually adjusted for each subject within a pilot trial prior to the measurements. Thereby, the lean angle – defined as the angle between the vertical in the sagittal plane and the line connecting the CoM and the center of the ankle joint – was gradually increased until the subjects no longer felt able to recover balance by taking a single step. Once the lean angle was determined, this angle was maintained throughout all recovery trials. The mean angle of the forward lean was 36.15 ± 1.38°, evoking a mean horizontal force component of 29.65 ± 2.99% of the subject’s body weight; which is very similar to the loads used by Karamanidis et al. (2008), Barrett et al. (2012), and Carty et al. (2012).

In this position, with heels touching the ground and arms hanging at their sides, the subjects were asked to concurrently perform the oral motor tasks. The respective bite force had to be maintained for at least 2 s until the investigators randomly released the electromagnetic system within a timeframe of 5 s. Once the forward fall was initiated, the subjects were encouraged to restore balance by taking a rapid single step placing their recovery limb properly in front of their other limb.

After one familiarization trial, for each test condition five trials were conducted.

### Measurements

All data collected were simultaneously recorded by a Vicon motion capture system (Vicon Motion Systems; Oxford Metrics Group, Oxford, UK). As indicated above, bite forces were measured by means of a hydrostatic system, sampling at 1,000 Hz. Besides, kinematic data were captured by use of thirteen infrared cameras (Vicon MX camera system, 200 Hz) and 39 passive reflective markers (diameter 14 mm). The reflective markers were placed on the subjects’ skin in accordance with the Vicon Plug-In Gait full-body marker set. Based on this, mathematical human multibody models (Kadaba et al., 1990; Davis et al., 1991) allowed for the definition of rigid body segments and its CoM, and the calculation of kinematic parameters, such as joint angles.

### Data Analysis

As all participants were able to successfully recover balance with a single step, all five trials were included in the analyses based on which mean values were calculated for each test condition. Hereto, data were processed using MATLAB R2014b (The MathWorks, Natick, MA, USA).

Initially, the time series were filtered by use of a fourth-order Butterworth low-pass filter with a cut-off frequency of 8 Hz. To determine the potential effects of FB on balance recovery, thereafter, for each trial four time points were identified (**Figure 1**): release of the subject (*Release*, 1A), toe-off from the ground of the recovery limb (*Toe-Off*, 1B), touchdown of the recovery limb (*Touchdown*, 1C), and 500 ms after touchdown (*TD_+*500*_*, 1D) (Karamanidis et al., 2008). For time-normalized analyses, additionally two main phases of recovery were defined: the *Falling phase* covered the time interval from *Release* until *Touchdown* (normalized from –100 to 0%), and the *Stance phase* involved the period between *Touchdown* and *TD_+500_* (normalized from 0 to 100%).

#### Spatiotemporal Parameters

Based on the abovementioned time points, subjects’ response time and duration of recovery were determined. The response time was considered as the time interval from *Release* until *Toe-Off*, and the duration of recovery was indicated by the time interval from *Release* until *Touchdown*. Further, the step length, defined as the linear distance between the initial and final toe position in anteroposterior direction, was calculated.

#### Joint Angles

Joint kinematics were analyzed in sagittal plane for the hip, knee, and ankle joints of the recovery limb. Specifically, mean joint angles at *Touchdown* and maximum joint flexion angles (dorsiflexion angle in terms of the ankle joint) during the *Stance phase* were investigated.

#### Dynamic Stability

Postural stability was quantified by the extrapolated CoM concept (Hof et al., 2005). This concept is based on the inverted pendulum model of balance and allows to determine postural stability in dynamic situations (Hof et al., 2005; Arampatzis et al., 2008; Hof, 2008; Curtze et al., 2010). Hereto, the margin of stability (MoS) in anteroposterior direction was calculated as it has been proposed by Hof et al. (2005):

MoS=pBoS−xCoM

equation

in which pBoS is the anterior boundary of the BoS (projection of the anteroposterior position of the toe from the recovery limb on the ground), and xCoM is the extrapolated CoM in the anteroposterior direction. The extrapolated CoM in turn was calculated as follows:

$$\text{xCoM}=\text{pCoM}+\frac{\text{vCoM}}{\sqrt{\text{g}/1}}$$

where pCoM is the anteroposterior component of the CoM (projection of the anteroposterior position of the CoM on the ground), vCoM is the anteroposterior velocity of the CoM, g is the acceleration of gravity, and l is the distance between the CoM and the center of the ankle joint in the sagittal plane (Hof, 2008).

The extrapolated CoM concept suggests that postural stability in anteroposterior direction is maintained when the projection of the extrapolated CoM is located within the BoS, i.e., the MoS shows positive values (Hof et al., 2005; Karamanidis et al., 2008). A loss of dynamic stability in turn is indicated by negative values of the MoS, i.e., in cases where the extrapolated CoM exceeds the anterior boundary of the BoS. The moment the MoS changed from negative to positive values (subsequently referred to as stability point), therefore was depicted as the main outcome parameter. In addition, dynamic stability was calculated for the moments of *Touchdown* and *TD_+*500*_*.

### Statistics

All statistical tests were performed by use of IBM SPSS Statistics 22.0 (IBM Corporation, Armonk, NY, USA). First, Kolmogorov–Smirnov tests were applied to confirm the normality of data distribution. Mauchly’s tests of sphericity were then conducted to determine whether the assumption of sphericity was violated. When this did occur, Greenhouse–Geisser estimates were used to correct for any violations.

Although the ordering was counterbalanced across the subjects, which minimized the likelihood of confounding, preliminary analyses were conducted to evaluate whether the order of exposure had an effect on the variables under investigation. However, repeated measures ANOVAs indicated that neither the order of presentation nor the trial number within both test conditions had been influential.

To check whether the subjects met the requested oral motor tasks, one-sample *t*-tests contrasted the intended and actual bite forces at subjects’ *Release* for both oral motor tasks. Differences in bite forces between oral motor tasks [FB, NB] and between time points [Release, Touchdown] were investigated by two-way repeated measures ANOVA. Finally, the effects of oral motor tasks on spatiotemporal parameters, joint angles, and dynamic stability were assessed by paired *t*-tests, separately run for each dependent variable under investigation.

All data are presented as mean values and 95% confidence intervals (mean ± CI_95%_). Effect sizes were determined using Cohen’s *d* (small effect: *d* = 0.20; medium effect: *d* = 0.50; large effect *d* = 0.80) or partial eta squared (small effect: $\eta_{p}^{2}=0.01$; medium effect: $\eta_{p}^{2}=0.06$; large effect $\eta_{p}^{2}=0.14$) in case of *t*-tests and ANOVAs, respectively (Cohen, 1988; Richardson, 2011). For all statistical tests, the level of significance was set *a priori* to *p* = 0.05.

## Results

### Bite Forces

**FIGURE 2:**
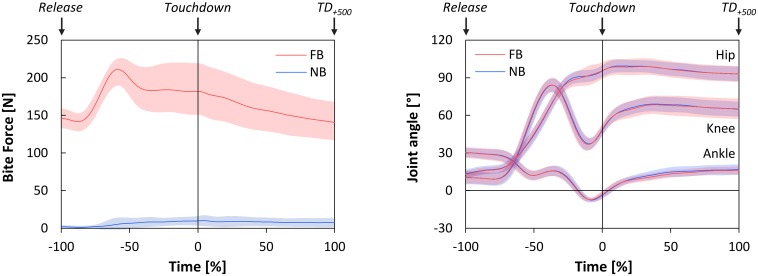
**Time-normalized bite forces **(left)**, and time-normalized joint angles of the hip, knee, and ankle joints in sagittal plane **(right)** during *Falling phase* and *Stance phase* as functions of oral motor tasks (FB, force-controlled biting; NB, non-biting)**. *Falling phase*: from *Release* until *Touchdown* (normalized from –100 to 0%); *Stance phase*: from *Touchdown* until 500 ms after Touchdown (*TD_+*500*_*) (normalized from 0 to 100%). All data are presented as mean ± CI_95_.

The time-normalized bite forces from *Release* until *TD_+500_* are shown in **Figure 2**. Descriptively, bite forces under FB conditions increased from 150 N at *Release* to over 200 N at *Toe-Off*, with a subsequent decrease to baseline values. But, also in NB slight increases in bite force from *Release* to *Toe-Off* and to *Touchdown* were observed.

Statistical tests revealed no significant deviations of the actual bite forces from the intended bite forces at *Release* [FB: *p* = 0.515; NB: *p* = 0.056], and thus confirmed the compliance with the oral motor tasks. On the other hand, significant main effects of oral motor tasks [*p* < 0.001, $\eta_{p}^{2}=0.97$] and time points [*p* = 0.006, $\eta_{p}^{2}=0.951$] were indicated by two-way repeated measures ANOVA. *Post hoc* analysis (paired *t*-tests) revealed that bite forces were statistically higher under FB as compared to NB, both at *Release* [*t*_(11)_ = 26.03, *p* < 0.001, *d* = 9.64] and at *Touchdown* [*t*_(11)_ = 12.92, *p* < 0.001, *d* = 4.82]. In addition, bite forces under both oral motor tasks significantly increased from *Release* to *Touchdown* [FB: *t*_(11)_ = 3.03, *p* = 0.011, *d* = 0.96; NB: *t*_(11)_ = 2.94, *p* = 0.014, *d* = 1.03].

### Spatiotemporal Parameters

Spatiotemporal parameters were analyzed to provide a general view on the effects of jaw clenching on balance recovery. However, the response time and the duration of recovery were both not significantly influenced by oral motor tasks. Moreover, there was no significant difference between the subjects’ step length in the two testing conditions (**Table 1**).

**Table 1:** Spatiotemporal parameters for force-controlled biting (FB) and non-biting (NB).

	FB	NB	*t_(11)_*	*p*	*d*
Response time [s]	0.162 ± 0.011	0.163 ± 0.013	0.30	0.770	0.05
Duration of recovery [s]	0.409 ± 0.019	0.414 ± 0.019	1.35	0.206	0.17
Step length [m]	1.086 ± 0.079	1.104 ± 0.080	1.70	0.118	0.15

*Mean ± CI_95%_.*

### Joint Angles

The time-normalized joint angles of the hip, knee, and ankle joints in the sagittal plane are illustrated in **Figure 2**. All joint angles at *Touchdown* were statistically unaffected by oral motor tasks. Additionally, maximum joint flexion angles in *Stance phase* showed no significant differences between FB and NB (**Table 2**).

**Table 2:** Joint flexion angles at *Touchdown*, and maximum joint flexion angles during *Stance phase* for force-controlled biting (FB) and non-biting (NB).

	FB	NB	*t_(11)_*	*p*	*d*
*Touchdown*					
Ankle angle [°]	–3.12 2.98	–3.64 2.90	0.89	0.395	0.11
Knee angle [°]	48.79 4.77	48.06 4.17	0.90	0.390	0.10
Hip angle [°]	95.68 6.71	95.35 4.71	0.20	0.849	0.04
*Stance phase*					
Maximum ankle flexion angle [°]	17.77 2.71	18.24 4.04	0.32	0.755	0.09
Maximum knee flexion angle [°]	69.96 7.60	70.15 5.82	0.14	0.892	0.02
Maximum hip flexion angle [°]	101.07 6.31	101.52 4.94	0.35	0.736	0.05

*Mean ± CI_95%_. Negative values for ankle joint angle represent a dorsi flexion, and positive values indicate a plantar flexion, respectively.*

### Dynamic Stability

**FIGURE 3:**
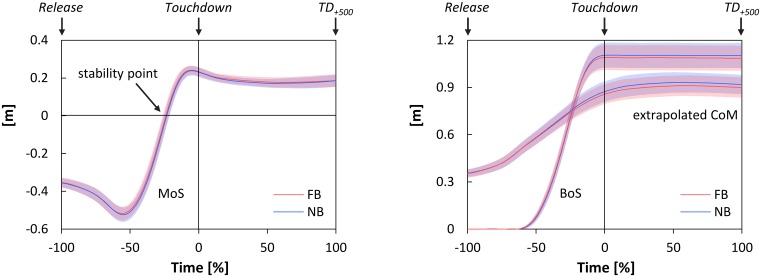
**Time-normalized data of the margin of stability (MoS, **left)**, the extrapolated center of mass (extrapolated CoM), and the anterior boundary of the base of support (BoS, both **right)** during *Falling phase* and *Stance phase* as functions of oral motor tasks (FB, force-controlled biting; NB, non-biting).** *Falling phase*: from *Release* until *Touchdown* (normalized from –100 to 0%); *Stance phase*: from *Touchdown* until 500 ms after Touchdown (*TD_+*500*_*) (normalized from 0 to 100%); stability point: time point the MoS changes from negative to positive values. All data are presented as mean ± CI_95_.

**Figure 3** shows the time-normalized data of the MoS, the extrapolated CoM, and the anterior boundary of the BoS. The stability point was obtained at -23.88 ± 2.33% and -23.22 ± 2.21% of the falling phase for FB and NB, respectively. In particular, paired *t*-test indicated no significant difference in this point between testing conditions [*t*_(11)_ = 1.48, *p* = 0.168, *d* = 0.19]. Further, FB did not significantly affect the anterior boundary of the BoS, the anteroposterior component and velocity of the CoM, the extrapolated CoM, and the MoS; neither at *Touchdown* nor at *TD_+*500*_* (**Table 3**).

**Table 3:** Stability parameters at *Touchdown* and 500 ms after Touchdown (*TD_+500_*) for force-controlled biting (FB) and non-biting (NB).

	FB	NB	*t_(11)_*	*p*	*d*
*Touchdown*					
Boundary BoS [m]	1.090 0.080	1.105 0.080	1.44	0.177	0.12
Position CoM [m]	0.731 0.053	0.742 0.054	1.63	0.131	0.14
Velocity CoM [m/s]	0.501 0.056	0.521 0.059	1.59	0.141	0.23
Extrapolated CoM [m]	0.857 0.063	0.874 0.065	1.80	0.100	0.17
MoS [m]	0.234 0.024	0.231 0.025	0.39	0.701	0.06
*TD_+*500*_*					
Boundary BoS [m]	1.086 0.079	1.104 0.080	1.70	0.118	0.15
Position CoM [m]	0.902 0.065	0.922 0.062	1.12	0.286	0.20
Velocity CoM [m/s]	–0.010 0.018	–0.012 0.021	0.20	0.842	0.08
Extrapolated CoM [m]	0.902 0.065	0.922 0.062	1.12	0.286	0.20
MoS [m]	0.184 0.030	0.182 0.028	0.13	0.900	0.03

*Mean ± CI_95%_. BoS, base of support; CoM, center of mass; MoS, margin of stability.*

## Discussion

The aim of the present study was to examine the effects of submaximum jaw clenching on dynamic postural stability and joint kinematics during balance recovery after forward loss of balance. We hypothesized that force-controlled biting would lead to improved balance recovery in terms of increased dynamic stability and lower joint flexion angles of the knee and hip at touchdown and during the subsequent stance phase. The results, however, showed that biting at a submaximum force did not significantly influence recovery behavior of healthy young adults with regard to the variables under investigation.

Previous studies on the impact of concurrent jaw clenching activities observed significant improvements in peak force and rate of force development as compared to non-clenching controls (Forgione et al., 1991; Hiroshi, 2003; Ebben et al., 2008). Further, significant reductions in CoP displacements have been described under static conditions (Hellmann et al., 2011; Ringhof et al., 2015b). To the authors’ knowledge, this study was the first to examine the effects of oral motor activities on dynamic postural stability in general, and specifically on balance recovery from forward loss of balance. Nevertheless, the present data are very similar in magnitude to those of other studies on balance recovery (Karamanidis and Arampatzis, 2007; Karamanidis et al., 2008; Curtze et al., 2010). This forms the basis for the further discussion, enabling conclusive statements with regard to dynamic postural stability. In this context, the authors attempt to provide some explanations for the lack of observed differences; without any claim to be comprehensive.

First, the absence of any biting effects might be evoked by the different motor demands associated with static postural control compared with balance recovery after simulated forward falls. The former is primarily based on fine motor control relying on feedback mechanisms, and unconscious and highly automated processes (Horak, 2006). Contrastingly, the latter requires gross motor coordination, and huge demands on explosive muscle activation and force production (Karamanidis et al., 2008). Specifically, in the scenario of simulated forward falls, this process mainly follows feedforward control. Hence, the subjects can preselect their compensatory movements, which finally reduces the contribution of reflexes and automated processes. This distinction is of particular relevance, because most effects of jaw clenching activities were considered to be caused by facilitation of reflexes and motor system excitability (Miyahara et al., 1996; Boroojerdi et al., 2000; Takada et al., 2000). Modulation of somatosensory input, particularly for the neck muscles (Abrahams, 1977) during simulated forward falls might, thus, be not an issue.

In this context, one could speculate that more ecologically valid experiments, which are representative for the analysis of real falls, might have revealed differing results. As indicated above, in simulated forward falls, the subjects are well aware of the upcoming forward fall, which increases the proportion of voluntary movement control. In everyday life, however, the subjects are mostly unaware of such tripping or slipping events. The process of recovering stability, hence, mainly follows stereotypic movement patterns, initially provoked by stimulation of the muscle spindles in the calf muscles. Consequently, recovery is primary based on reflexes or automated compensatory movements, all requiring no or only little focused attention (Macpherson and Horak, 2013). Based on the findings on reflex facilitation (Miyahara et al., 1996; Takada et al., 2000) and concurrent activation potentiation (Ebben, 2006), we hypothesize that investigation on unexpected perturbations possibly would offer ergogenic effects for concurrent clenching activities, whereas the increase in voluntary movement control evoked by the chosen study design might have contributed to the absence of any significant alterations.

On the other hand, the lack of observed differences in dynamic stability could be the result of a floor effect, whereby the perturbation for the given sample was not difficult enough. Subjects were young healthy adults, and all obtained very high stability values (MoS > 0.2 at *Touchdown*). Potentially, subjects with diminished postural control and/or reduced force potential as, e.g., elderly could benefit from force-controlled biting. This investigation cannot resolve this question, however.

The final factor to be considered in the interpretation of the data is the bite force. At *Release* bite forces under both testing conditions were maintained at the intended level, confirming the compliance with the oral motor tasks. At *Touchdown*, however, significant increases in bite forces for both oral motor tasks were observed. In terms of NB, the results imply that an open-mouth, non-clenching condition is an unphysiological state which is not preferred during challenging situations. This is reinforced additionally by the fact that the subjects, even when already submaximum clenching their jaw, significantly increased their bite force from 146.01 ± 13.04 N to more than 200 N at *Toe-Off*. Clenching the jaw, hence, seems to be part of a common physiological repertoire used to improve the neural drive to distal body segments and, by this means, to enhance performance in many ways (Ebben, 2006; Ringhof et al., 2015a). This, in turn, would suggest that many studies focusing the ergogenic effects of jaw clenching actually did not observe performance improvements when the jaw was clenched, but rather a decrease in the non-clenching condition (Hiroshi, 2003; Ebben et al., 2008).

In conclusion, the present study has shown that submaximum clenching the jaw did not significantly affect balance recovery of healthy young adults in terms of dynamic postural stability and lower extremity joint kinematics after forward loss of balance. This is probably due to the different control strategies associated with static postural control and balance recovery after simulated forward falls. One must, therefore, question whether (i) the ergogenic effects of jaw clenching are limited only to static postural control, or (ii) the contradictory results might have been evoked by the methodological approach, the bite forces itself, or the study sample. Conclusive evidence is lacking, however. On the other hand, one might argue that reductions in CoP displacement – as they have been observed in response to jaw clenching (Hellmann et al., 2011; Ringhof et al., 2015b), and centric jaw relation (Gangloff et al., 2000; Bracco et al., 2004; Sakaguchi et al., 2007) – could degrade postural control performance. According to Haddad et al. (2013), postural variability in terms of increased CoP displacements is considered to aid in the exploration of the environment and to allow to experience the boundaries of stability (van Emmerik and van Wegen, 2002). However, this explanatory behavior might be valid only as long as postural sway does not cause a loss of balance, and rather may facilitate postural control during postural perturbation, when increased CoP displacement may make it easier to regain balance. For static postural control, this assumption, therefore, should be regarded critically; particularly, as this explanatory behavior increases the risk of losing balance by decreasing the MoS.

Future studies should contrast the effects of submaximum biting in different populations, under conduction of random perturbations, and as compared to both open mouth and habitual control conditions.

## Author Contributions

The corresponding author confirms that all authors were fully involved in the study and preparation of the manuscript process. Each of the authors has read and approved the final version of the manuscript, and all authors agreed to the submission to Frontiers in Psychology.

## Conflict of Interest Statement

The authors declare that the research was conducted in the absence of any commercial or financial relationships that could be construed as a potential conflict of interest.
